# Genetic and epigenetic divergence among *Arabidopsis thaliana* Col-0 laboratory lineages since the 1950s

**DOI:** 10.64898/2026.08.05.743031

**Published:** 2026-08-06

**Authors:** Alexandra M. Tadros, Zhilin Zhang, Nan Yao, Rebecca A. Mosher, Frank Johannes, Robert J. Schmitz

## Abstract

The model plant *Arabidopsis thaliana* has been a workhorse of plant biology since the 1950s, when the Columbia-0 (Col-0) accession was widely adopted by the research community as a reference genotype. Since then, Col-0 seeds have been shared among laboratories worldwide and propagated independently, creating a network of related laboratory lineages. Here, we sequenced the genomes and DNA methylomes of 78 Col-0 strains from laboratories and stock centers around the world to assess the genetic and epigenetic variation accumulated during this period and to reconstruct their propagation history from *de novo* variants. After quality filtering, we identified 1,415 single-nucleotide polymorphisms and 169,677 epimutations across 75 Col-0 lines and used these variants to generate SNP- and epimutation-based phylogenies. The two phylogenies showed highly concordant topologies, but the epimutation-based tree provided greater resolution of relationships among closely related lineages. Divergence-time estimates suggest that many lines have been propagated for approximately one generation per year over several decades, resulting in pairwise divergences of up to approximately 100 generations. We further identified 170 nonsynonymous substitutions and more than 200 candidate epialleles, some of which were associated with gene-expression differences among Col-0 clades and may therefore have functional consequences. Together, these findings show that Col-0 is best viewed not as a single invariant reference genotype, but as a collection of related laboratory lineages that have accumulated genetic, epigenetic, and transcriptional divergence through time, with potential consequences for the reproducibility of experiments across laboratories.

**Significance Statement:**

The *Arabidopsis* Col-0 reference accession is often treated as a single, invariant genotype, yet independently maintained laboratory stocks have accumulated substantial genetic, epigenetic, and transcriptional divergence since the 1950s. Some lineages are separated by more than 100 generations of independent propagation. Mutations and CG epimutations reconstruct nearly identical lineage histories, demonstrating that heritable DNA methylation changes provide a robust record of recent laboratory evolution.

## Full Text Availability

The license terms selected by the author(s) for this preprint version do not permit archiving in PMC. The full text is available from the preprint server.

